# Factors affecting the availability of tracer health commodities in public facilities at Tana River County, Kenya

**DOI:** 10.1186/s40545-023-00658-6

**Published:** 2023-11-15

**Authors:** Japheth Araka Ayako, Peter Ndirangu Karimi, Eugene Rutungwa, Joseph Lune Ngenzi, Kelvin Wangira Nyongesa, Racheal Habona Jillo, Miheso Hassler Kavere, Abdul Galgalo Shambaro

**Affiliations:** 1 EAC Regional Centre of Excellence for Vaccines, Immunization and Health Supply Chain Management, College of Medicine and Health Sciences, University of Rwanda, Kigali, Rwanda; 2 Tana River County Government (004), Mombasa, Kenya; 3 Faculty of Health Sciences, University of Nairobi, Nairobi, Kenya; 4 School of Business, College of Business and Economics, University of Rwanda, Kigali, Rwanda; 5 School of Public Health, College of Medicine and Health Sciences, University of Rwanda, Kigali, Rwanda

**Keywords:** Tracer commodities, Stock control cards, Availability, Public health facilities, Tana River County

## Abstract

**Background:**

Delivery of quality healthcare is significantly based on the level of commitment among health facilities. This includes building a strong system with the continued availability of tracer commodities. Human resources, financing, health information provision, and technologies integrated into the care environment have been vital in defining improved care.

**Methods:**

This was a cross-sectional study conducted in health facilities in Tana River County. A census method was used where all 62 health facilities across different tiers of healthcare delivery were considered. Out of 62 facilities, 60 participated in the study. A structured questionnaire and a checklist were used to collect data. Data were analysed using both descriptive and inferential statistics at 0.05 level of significance. Statistical Package for Social Sciences version 26 was used for data analysis.

**Results:**

Majority of the participants were nurses (71.7%), male (68.3%), and diploma holders (78.3%). The mean availability of the tracer commodities was 68.73%. The human resource-related factors influencing availability were personnel training on commodity management (β = 4.56, 95%CI 2.29–11.21, *p* = 0.012) and presence of pharmaceutical technicians dispensing commodities (β = 2.85, 95%CI 1.29–5.21, *p* = 0.005) Financial factors investigated revealed that those who were in county hospitals (β = 19.11, 95%CI 7.39–30.83, *p* = 0.002) and facilities which has disbursement of budgetary allocation on time (β = 12.08, 95%CI 3.11–23.57, *p* = 0.002) had higher availability of tracer commodities.

**Conclusion:**

There was moderate availability of tracer commodities which was influenced by training, personnel, level of the facility, and budget allocation on time.

## Background

The availability of tracer health commodities is essential in a healthcare setting and defines the delivery of quality services. Tracer drugs are representative of essential medicines and satisfy the priority healthcare needs of a given population [1]. The key factors defining healthcare delivery include human resources, health financing, information technologies, leadership, and governance [2].

Globally, the WHO action recommends the availability of at least 80% availability of tracer health commodities in health facilities to provide quality care [3]. Approximately 30–50 percent of the global population does not have access to essential medicines [4–6]. In Central African Countries, an analysis of ten important medications indicated insufficient commodity supply at all levels of the health system. To investigate supply chain characteristics that influence medication availability in sub-Saharan Africa, a project termed supply chains for community case management was created [7]. The availability of tracer medicines in sub-Saharan Africa has been investigated and found to be inadequate in several countries [1, 8, 9]. Factors associated with drug availability include drug requisitions, poor logistics distribution, the push of medical products, distribution delays, supplying essential medicines with short expiry dates, and low-usage drugs [10].

In Kenya, the decentralisation of healthcare services has seen a focus on service delivery within the County context, bringing a different dimension to the availability of essential commodities [11]. Patients accessing health facilities increased when Kenya devolved health services and introduction of UHC, which varies from one county to another. This is because of different leadership and governance styles, allocation of funds, procurement practices, human resource capacity, and commodity management practices in different counties [12]. To treat common diseases, there is a need to ensure that tracer health commodities are available to the patients, at the right time and place to reduce morbidity and mortality [13]. From previous studies done in Kenya, none has been done in Tana River County. During the quantification exercise, it was observed from the stock control cards that there were frequent stockouts of medicine in public health facilities [14]. Tana River County has very minimal private facilities hence on average. Patients walk more than 20 km to access health services and therefore they are not supposed to miss tracer commodities once they visit the facility [14].

This study investigated the availability of tracer commodities within Tana River County and identified the underlying challenges to avoid stockouts.

## Methodology

### Study area and period

The study was conducted from November 2022 to January 2023 within Tana River County health facilities, in the Republic of Kenya. The county is arid and semi-arid and has a total area of 38,862 square kilometres. It has 62 health facilities, including one County Referral Hospital, two Sub-County Hospitals, four Health Centres, and 55 dispensaries [11]. From the World Bank report on quantification there were frequent stockouts of tracer commodities, but the reasons for this situation were not identified in Tana River County.

### Study design

The study design used was cross-sectional. This design was appropriate because it provided an understanding by comparison and collaboration of the relevant documents and data from the facility managers about the availability of tracer essential health commodities at one point in time.

### Study population and sampling

The study population comprised all 62 health facilities within the county. A census method was used where all 62 health facilities across different tiers of healthcare delivery were considered. Out of 62 facilities, 60 participated in the study. The facility in-charges who were the overall facility managers of commodity management, were the key respondents. Census sampling means all these health facilities are owned by the government or Faith Based Organisations.

### Data collection tools

The study adopted a structured research questionnaire and an observational checklist. The structured questionnaire included both open and closed-ended questions. A checklist was also used to observe tracer commodities availability in each of the facilities investigated. The questionnaire used in the study had four sections. Section one collected information about the characteristics of the individuals in charge of the facilities being studied. Section two focused on the availability of tracer health commodities. Section three explored human resource-related factors and section four examined finance-related factors. Additionally, a checklist was used to observe the availability of tracer commodities in each facility.

### Data collection procedure

Approval was sought from the relevant authority for the study. The research assistants were trained on how to collect the data. They visited the health facilities and sought permission from the management and explained the purpose of the research. They then proceeded to the participants and requested them to consent. The respondents were requested to fill out the questionnaires and provide any other relevant data.

The person in charge of the pharmacy was requested to provide data on the availability of tracer commodities. The collection of data from the bin cards was done using the checklist and asking the necessary questions. The data on the human resource factors were collected from the person in charge of the health facilities. The filled questionnaires were collected concurrently for further processing. The filled tools were kept in a secured cabinet to ensure confidentiality.

### Data analysis

The collected data were entered, cleaned, and coded using Microsoft Excel 2016 before the analysis. It was then uploaded to Statistical Package for Social Sciences version 26. Both descriptive and inferential statistics were used. Descriptive statistics included mean, range, standard deviation and proportions. Inferential statistics used linear regression analysis to establish the relationship between availability of medicines and other variables at 0.05 level of significance.

## Results

### Sociodemographic characteristics

The sociodemographic characteristics are summarised in Table 1. The average age of the respondents was 33.48 (SD ± 6.6) years and the range was 24 to 58 years. The overall participants’ mean work experience was 6.8 (SD ± 5.95) years, and in their respective health facilities was 3.53 (SD ± 3.02) years. The majority of the respondents were male (41, 68.33%), and nurses predominated (43,71.67%). Most (47,78.3%) of the participants were diploma holders.

**Table 1:** Sociodemographic characteristics (*n* = 60)

Characteristics	Categories	Frequency	Percentage
Gender	Male	41	68.3
	Female	19	31.7
Job title	Community health volunteer	3	5.0
	Pharmacy technician	7	11.7
	Nurse	43	71.7
	Clinical officer	7	11.7
Education level	Diploma	47	78.3
	Higher diploma	4	6.7
	Degree	3	5.0
	Certificate	6	10.0

### Availability of tracer health commodities

The mean availability of the tracer commodities was 68.73% with a standard deviation of 14.41 and a range of 27% to 94.6%. Fifty-seven (95%) facilities had stock control cards in place (Table 2). Fifty-four (90%) were using the stock cards and 40 (66.67%) had them updated. The average lead time was 39 days with a standard deviation of 10.7 days and a range of 22 to 90 days. The fill rate was at 56.8% and the range of 30% to 70% while the standard deviation was 10.3 days. All the facilities in the county used the manual system of commodity management.

**Table 2:** Availability of tracer health commodities (*n* = 60)

Tracer commodity	Frequency	Percent
Adrenaline injection 1 mg/ml	24	40
Albendazole Tab. 400 mg	59	98.33
Amoxicillin capsules, 250 Mg/500 Mg	20	33.33
Amoxicillin dispersible tablets, 250 mg	49	81.67
Benzylpenicillin injection 1 MU OR 5 MU	46	76.67
Chlorhexidine gel, 7.1% (as digluconate) (20 g tube)	51	85
Gentamicin injection, 40 mg/2 ml	25	41.67
Hydrocortisone injection 100 mg	59	98.33
Magnesium sulphate injection, 500 mg/mL (50%), 10 mL	20	33.33
Metronidazole tablet, 400Mgs/200Mgs	29	48.33
Nystatin oral suspension 100 IU/ml	47	78.33
ORS Co-Pack (4 sachets of low osmolarity ORS (500 ml formulation) + 10 tablets of dispersible zinc sulfate tablets 20 mg)	55	91.67
Oxytocin injection 10 I.U	46	76.67
Paracetamol syrup/suspension, 120 mg/5 ml	40	66.67
Paracetamol tablets, 500 mg	33	55
Sodium chloride, 0.9% (isotonic), (500 mL bottle)	52	86.67
Sodium hypochlorite solution 4–6%	39	65
Tetracycline eye ointment, 1%, 3.5 g tube	42	70
Autoclaving tape, 3/4’’	45	75
Catheter, Foley’s, 18FG 30 mL 2-way	38	63.33
Cord clamp	52	86.67
Cotton gauze plain, 36" x 100yds, 1,500 g	58	96.67
Cotton wool, absorbent, 400 g	49	81.67
Gloves, latex, examination, medium	58	96.67
Gloves, surgical, size 7.5 (sterile)	36	60
IV cannula 18G	48	80
IV cannula 20G	43	71.67
Maternity pad, 26 cm x 9 cm x 1 cm	51	85
Nasal prongs for oxygen delivery, adult size	5	8.33
Nasal prongs for oxygen delivery, paediatric size	6	10
Solusets for fluids	27	45
Surgical blade with handle, size 23	42	70
Suture nylon No.2/0,3/8 Circle, 45 mm, 100 cm, RCN	46	76.67
Suture polyglactin 2/0 75 cm on 40 Mm ½ Circle RBN	27	45
Syringe 2 mL + needle 23G × 1"	53	88.33
Syringe 5 mL + needle 21G × 1.5"	56	93.33
Zinc oxide strapping, 7.5 cm × 4.5 m	56	93.33

### Factors affecting the availability of tracer health commodities

#### Human resource factors

The findings revealed that 27 (45%) respondents had trained in commodity management in the last 6 months while 36 (60%) had trained in commodity management since employment (Table 3). Half of the participants acknowledged that the training was very relevant. Twenty-nine (48.3%) of them had been trained 3–4 times and 22 (61.1%) had stayed for 3–4 years since they were last trained at the time of the study. The majority (53, 88.33%) of those who dispensed health commodities were community health volunteers.

**Table 3:** Human resource factors influencing the availability of tracer commodities

Human resource factor	Frequency	Percent
Trained in commodity management in the last 6 months	27	40
Trained in commodity management since employment	36	60
Duration since the last training		
1–2 years	9	25.0
3–4 years	22	61.1
5 years and above	5	13.9
Number of times participants had been trained		
3–4 times	29	48.3
More than 5 times	13	21.7
1–2 times	18	30.0
Qualification of the person dispensing health commodities		
Pharmaceutical technicians	7	11.7
Community healthy volunteer	53	88.3

#### Financing-related factors

Different categories of the level of care influenced the amount of money received for purchases of tracer commodities. As indicated in table 4, fifty-three (88.33%) facilities were Level Two which are also called Dispensaries while four (6.67%) were Level 3 also known as Health centres. In addition, two (3.33%) were level 4 facilities also called sub-county hospitals and there was one (1.67%) level 5 also known as the County Referral Hospital. The average amount allocated to each facility for the purchase of commodities was Ksh 679,592. The range was from Ksh 211,230 to Ksh 2,867,794. The standard deviation was Ksh 663,874 which shows a wide variation in the amount allocated to these health facilities. No facility received an adequate budgetary allocation for the purchase of health commodities and the disbursement was done half yearly. Out of the facilities that participated in the study 55 (91.7%) received a budgetary allocation out of which only 5 (8.33%) indicated it was adequate.

**Table 4:** Finance-related factors influencing the availability of tracer commodities

Factor	Frequency	Percent
Level of care facility		
Dispensary	53	88.33
Health centre	4	6.67
Sub county hospital	2	3.33
County referral	1	1.67
Budgetary allocation adequacy	5	8.33
Receiving budgetary allocation	55	91.7

#### Predictors of the availability of tracer commodities

Inferential statistics was employed to establish the relationship between availability of tracer commodities as the dependent variable. The independent variables were human and financing related factors. Linear regression was used because the dependent variable is continuous. The level of significance was set at 0.05.

Analysis was conducted to investigate the association between the human resource variables and the availability of medicines. Availability of tracer commodities was 4.56 times higher in facilities that had personnel trained in commodity management since employment (β = 4.56, 95%CI 2.29–11.21, *p* = 0.012). The findings also revealed that facilities that had pharmaceutical technicians dispensing commodities had 2.85 times increase in the availability of tracer commodities (β = 2.85, 95%CI 1.29–5.21, *p* = 0.005) compared to those without.

The relationship between finance factors and availability of tracer commodities showed that county and sub-county-level health facilities had higher availability of tracer commodities (β = 19.11, 95%CI 7.39–30.83, *p* = 0.002) compared to the lower-level facilities. The availability of tracer health commodities was found to be 12.08 times more in those facilities that had disbursement of budgetary allocation on time (β = 12.08, 95%CI 3.11–23.57, *p* = 0.002).

The variables that were found to be statistically significant at the bivariate level were subjected to multiple linear regression analysis to determine the independent predictors of the availability of tracer commodities. Training in commodity management since employment and level of health facilities were found to be the significant factors. The availability of tracer health commodities was 3.22 more in facilities that had dispensing personnel who had been trained in commodity management since employment (β = 3.22, 95%CI 1.45–11.61, *p* = 0.002). Higher level health facilities had higher availability of tracer commodities (β = 20.52, 95%CI 4.25–36.79, *p* = 0.014) compared to lower levels.

## Discussion

This study assessed the availability of tracer health commodities in Tana River County. The region experienced frequent stockouts according to the World Bank quantification report [15].

### Availability of tracer commodities

The overall availability of these commodities was at 68.73% compared to the recommended 80% by the WHO. The availability of these commodities was moderate considering their use in a hospital setting. These findings are comparable to a study conducted in Ethiopia [16]. Most health facilities have moderate amounts of tracer commodities for several reasons. They include limited funding, limited storage space, limited demand, and supply chain challenges. Therefore, health facilities must balance the need to have enough tracer commodities on hand with the need to operate within budget constraints and storage limitations. The availability of tracer commodities was lower compared to those from another study in Ethiopia [1] which shows better overall mean availability, mean duration, and average frequency of stock out of tracer drugs. On average, the availability of bin cards was at 95% which was higher [1]. These findings show that records are vital for health facilities and patient care. Stock control cards are used in hospitals to track the movement of health commodities. Having these stock control cards in facilities is crucial for quality care. The findings were lower compared to another study in Ethiopia where the availability of essential drugs was 91% [14]. The factors that impact the availability of these commodities are complex and multifaceted. They include funding, infrastructure, the availability of trained healthcare professionals, the cost of commodities, political and social factors. It is essential to address these factors to ensure that all people, regardless of their income level, have access to the high-quality medical care.

### Human resource factors associated with the availability of tracer commodities

Institutions that had healthcare personnel trained in commodity management had a high level of availability of these tracer commodities. Training promotes individual knowledge level and ability to make informed choices relating to the procurement of these commodities. These findings are comparable to a report published by the World Health Organization which reveals that global scarcity of qualified health workers limits the general accomplishment of health and development goals [2]. The report further showed that health workers are usually undertrained, underpaid, and lack resources, which makes them unmotivated [2]. There was lack of qualified pharmacy professionals to manage medicines and supply chains [17]. A study in Kenya that investigated the availability of tracer medicines established that the lack of training of healthcare workers in commodity management affected their availability [12].

The cadre of healthcare professionals dispensing medicines was a key predictor of the availability of tracer commodities. In health facilities where pharmaceutical technicians were responsible for dispensing tracer commodities, the availability was higher. These findings are consistent with those from Uganda which established that the availability of tracer commodities was influenced by the personnel handling them [18]. The lack of skilled health professionals in commodity supply chain management is a major barrier to the availability of vital medications in public health institutions [19].

### Finance-related factors affecting the availability of tracer health commodities

The level of the health facilities influenced the availability of tracer commodities with county hospitals showing a higher availability of tracer commodities. The level of hospital defines the urgency of care which outlines the greater need for tracer commodities. A study done in Nyeri County found that lower-tier hospitals had lower availability of tracer commodities compared to higher-tier hospitals [12]. The prioritisation of high-level hospitals was mainly informed by high emergency cases and the fact that they also act as referral facilities [12]. The high-level facilities had more tracer commodities than lower-level facilities [16]. Facilities that received budgetary allocation on time had more tracer commodities. A study in Kenya found that inadequate financing is a major significant factor associated with the availability of tracer commodities [12]. The same study revealed that there was a substantial link between the budget allocated and the availability of tracer commodities [12]. Budgetary allocation was statistically significantly associated with the availability of tracer drugs [12]. Without adequate funding, hospitals may struggle to procure tracer commodities and other necessary resources to support medication safety programmes.

## Limitations

The targeted facilities were only in the public sector. The private sector also offers the same services and they procure and manage their supplies hence that may give a different picture when investigated. The cross-sectional research design also provided a snapshot of the situation which may not be the case if conducted at a different point in time. Lastly, the study focused on the health facilities rather than service providers who may provide contrary opinions at the same facility.

Kenya healthcare system is devolved to the 40 regions called counties. These areas share similar challenges in providing adequate healthcare services. The results of this study will provide an objective insight on what requires to be done to realise quality services as the government endeavours to rollout universal health care for all. Developing countries face similar challenges and therefore findings from this study may be used to develop strategies in other countries to improve healthcare delivery.

## Conclusions

The availability of tracer health commodities was moderate. The factors that influenced their availability were training of the health personnel on commodity management, level of facility, timely budget allocation, and presence of pharmaceutical technician.

## Data Availability

The data used to generate this manuscript are available upon request to the corresponding author.

## Abbreviations

WHO: World Health Organization
CHV: Community Health Volunteer
SD: Standard deviation
CI: Confidence interval
KNH-UoN: Kenyatta National Hospital/University of Nairobi Ethics Review Committee
NACOSTI: National Commission for Science, Technology, and Innovation
